# Supplementary material for: Identification of photoperiod-induced specific miRNAs in the adrenal glands of Sunite sheep (Ovis aries)
Source: Front Vet Sci. 2022 Jul 22;9:888207. doi: 10.3389/fvets.2022.888207 (PMC9354845; doi:10.3389/fvets.2022.888207)
Supplement: Supplementary file 1 [file Data_Sheet_1.ZIP › Supplementary file/miRDeep2 file.zip!/miRDeep2 file/Know_Structure/SPLP42A1/oar-mir-494.pdf]

miRBase precursor : oar-mir-494
Total read count : 18247
oar-miR-494-5p read count : 53
oar-miR-494-3p read count : 18191
remaining reads : 3

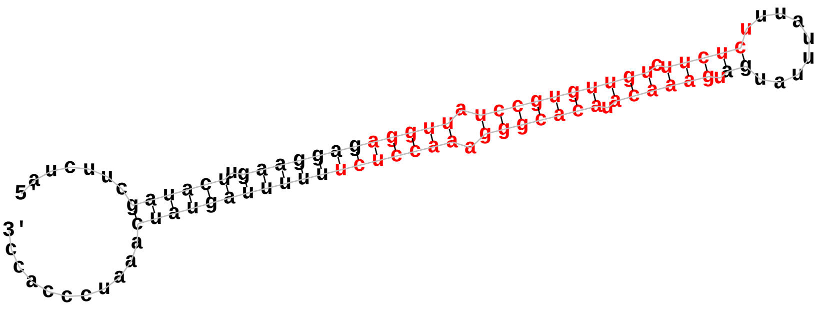

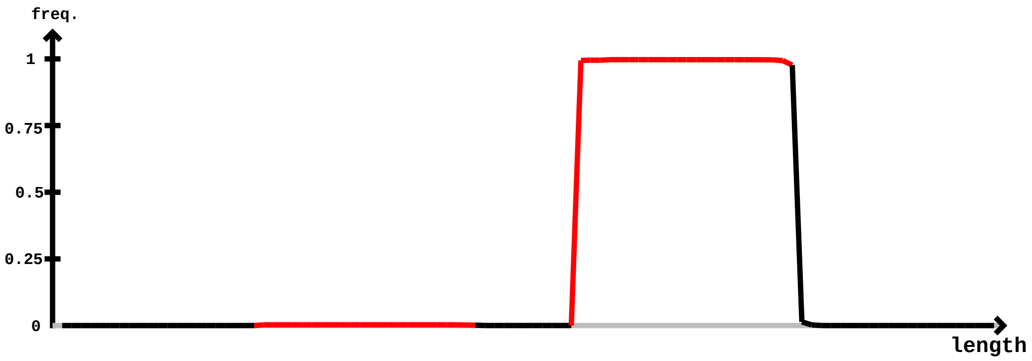


```
                                                 oar-miR-494-3p
                     oar-miR-494-5p
5'- aucuucgauacuugaaggagagguuauccguguugucuucucuuuauuuaugaugaaacauacacgggaaaccucuuuuuuaguaucaaaucccacc   -3'    exp
    ......(((((.((((((((((((.((((((((((.(((((.........)).))))))).))))))).))))))))))))))))))..........   reads      mm         sample
    ..................agagguuauccguguugucuu..........................................................   1          0          seq
    ....................agguuauccguguugucuucu........................................................   13         0          seq
    ....................agguuauccguguugucuucuc.......................................................   12         0          seq
    ....................agguuauccguguugucuucucu......................................................   24         0          seq
    ....................agguuauccguguugucuucucuu.....................................................   1          0          seq
    ....................agguuauccguguugucuucucuuua...................................................   1          0          seq
    ..........................uccguguugucuucucuuuau..................................................   1          0          seq
    .............................................auuuaugaugaGacaua...................................   2          1          seq
    ................................................uaugaugaaacauacacgggaaaccucu.....................   1          0          seq
    .....................................................ugaaacauacacgggaa...........................   1          0          seq
    .....................................................ugaaacauacacgggaaa..........................   1          0          seq
    .....................................................ugaaacauacacgggaaac.........................   4          0          seq
    .....................................................ugaaacauacacgggaaacc........................   6          0          seq
    .....................................................ugaaacauacacgggaaaccu.......................   49         0          seq
    .....................................................ugaaacauacacgggaaaccuc......................   304        0          seq
    .....................................................ugaaacauacacgggaaaccucu.....................   17555      0          seq
    .....................................................ugaaacauacacgggaaaccucuu....................   207        0          seq
    .....................................................ugaaacauacacgggaaaccucuuu...................   11         0          seq
    ......................................................gaaacauacacgggaaaccuc......................   1          0          seq
    ......................................................gaaacauacacgggaaaccucu.....................   15         0          seq
    ........................................................aacauacacgggaaaccucu.....................   1          0          seq
    ........................................................aacauacacgggaaaccucuuu...................   32         0          seq
    ........................................................aacauacacgggaaaccucuuuu..................   3          0          seq
    .........................................................acauacacgggaaaccucuuu...................   1          0          seq
```
